# Thymic epithelial tumors: examining the GTF2I mutation and developing a novel prognostic signature with LncRNA pairs to predict tumor recurrence

**DOI:** 10.1186/s12864-022-08880-3

**Published:** 2022-09-16

**Authors:** Wei Liu, Hao-Shuai Yang, Shao-Yi Zheng, Jian-Hao Weng, Hong-He Luo, Yi-Yan Lei, Yan-Fen Feng

**Affiliations:** 1 Department of Thoracic Surgery, The First Affiliated Hospital, Sun Yat-sen University, Guangzhou, 510080 Guangdong China; 2 State Key Laboratory of Oncology in South China, Collaborative Innovation Center for Cancer Medicine, Sun Yat-sen University Cancer Center, Guangzhou, 510060 Guangdong China; 3 Department of Pathology, Sun Yat-sen University Cancer Center, Guangzhou, 510060 Guangdong China

**Keywords:** GTF2I mutations, Immune infiltrates, lncRNA pairs, Thymic epithelial tumors, Tumor recurrence

## Abstract

**Background:**

General transcription factor IIi (GTF2I) mutations are very common in thymic epithelial tumors (TETs) and are related to a more favorable prognosis in TET patients. However, limited research has been conducted on the role of GTF2I in the tumor immune microenvironment (TIME). Further, long non-coding RNAs (lncRNAs) have been associated with the survival of patients with TETs. Therefore, this study aimed to explore the relationship between GTF2I mutations and TIME and build a new potential signature for predicting tumor recurrence in the TETs. Research data was downloaded from The Cancer Genome Atlas database and the CIBERSORT algorithm was used to evaluate TIME differences between GTF2I mutant and wild-type TETs. Relevant differentially expressed lncRNAs based on differentially expressed immune-related genes were identified to establish lncRNA pairs. We constructed a signature using univariate and multivariate Cox regression analyses.

**Results:**

GTF2I is the most commonly mutated gene in TETs, and is associated with an increased number of early-stage pathological types, as well as no history of myasthenia gravis or radiotherapy treatment. In the GTF2I wild-type group, immune score and immune cell infiltrations with M2 macrophages, activated mast cells, neutrophils, plasma, T helper follicular cells, and activated memory CD4 T cells were higher than the GTF2I mutant group. A risk model was built using five lncRNA pairs, and the 1-, 3-, and 5-year area under the curves were 0.782, 0.873, and 0.895, respectively. A higher risk score was related to more advanced histologic type.

**Conclusion:**

We can define the GTF2I mutant-type TET as an immune stable type and the GTF2I wild-type as an immune stressed type. A signature based on lncRNA pairs was also constructed to effectively predict tumor recurrence.

## Background

Thymic epithelial tumors (TETs) are tumors originating from thymic epithelial cells, with an incidence of approximately 1–3 cases/million, and are the most common type of anterior mediastinal tumor [1, 2]. According to the 2021 World Health Organization (WHO) classification, TETs can be classified into thymoma type A, AB, B1, B2, B3, and thymic carcinoma [3]. Type B2 and B3 thymoma and thymic carcinoma have a higher degree of malignancy compared with type A, AB and B1 thymomas, implying a less favourable prognosis [4, 5]. However, some studies have determined that type A, AB, and B1 thymomas with low grade oncological morphology were more likely to recur [6]. Masaoka stage has been identified as an independent prognostic factor with a strong ability to predict tumor recurrence and patient survival [7]. Nevertheless, neither Masaoka stage nor pathological classification are sufficiently accurate to predict tumor recurrence in TET patients, and there are no recognized biomarkers to predict TETs recurrence in clinical practice. General transcription factor IIi (GTF2I) mutations are reported at a high frequency in TETs, and these mutations have been associated with a favorable prognosis of patients with TETs [8, 9]. Recently, a growing number of studies have been conducted on tumor immune microenvironments (TIME) which increase our understanding of the immune mechanism during tumor development and metastasis, while also promoting the discovery of new methods for studying tumor development [10–12]. The relationship between GTF2I mutations and TIME has not previously been studied in TETs despite its importance to aid in understanding the molecular behavior of TETs to enable clinical application. Long non-coding RNA (lncRNA) are segments of non-coding RNA greater than 200 nucleotides in length that lack protein coding ability. An increasing number of studies are exploring the role of lncRNA during tumor development [13–15], for example, lncRNA H19 is a powerful prognostic biomarker of neuroendocrine prostate cancer that predicts the probability of tumor recurrence [16]. Several studies have also reported that lncRNA LOXL1-AS1, LINC00174, and XLOC_003810 play a key role in the development of TETs, which indicated the potential of lncRNA to predict the prognosis of TET patients [17–19]. As lncRNAs and GTF2I are both important in TETs, it is critical to determine whether lncRNAs associated with GTF2I mutations in TETs. Previous studies have established lncRNA signatures based on lncRNA expression levels to predict the prognosis of patients with TETs [20, 21]. However, the expression level of lncRNA depends on the detection platform; therefore, clinical utilization of these lncRNA signatures is limited by the accuracy of the detected lncRNA level. A prognostic gene or lncRNA signature using a gene or lncRNA pairs method has also been established, which is not restricted by the expression level of a gene or lncRNA and instead focuses on the expression level differences of a gene or lncRNA [22–24].

In this study, we first examined the TIME differences between GTF2I mutant and wild-type TETs based on The Cancer Genome Atlas (TCGA) patient data. Relevant differentially expressed lncRNAs were selected based on differentially expressed immune-related genes between GTF2I mutant and wild-type TETs. Next, we constructed a novel lncRNA signature to predict tumor recurrence in patients with TETs by applying the lncRNA pairs method.

## Results

### Characterizing TET mutations and GTF2I mutation relation to clinical features and survival

**Fig. 1:**
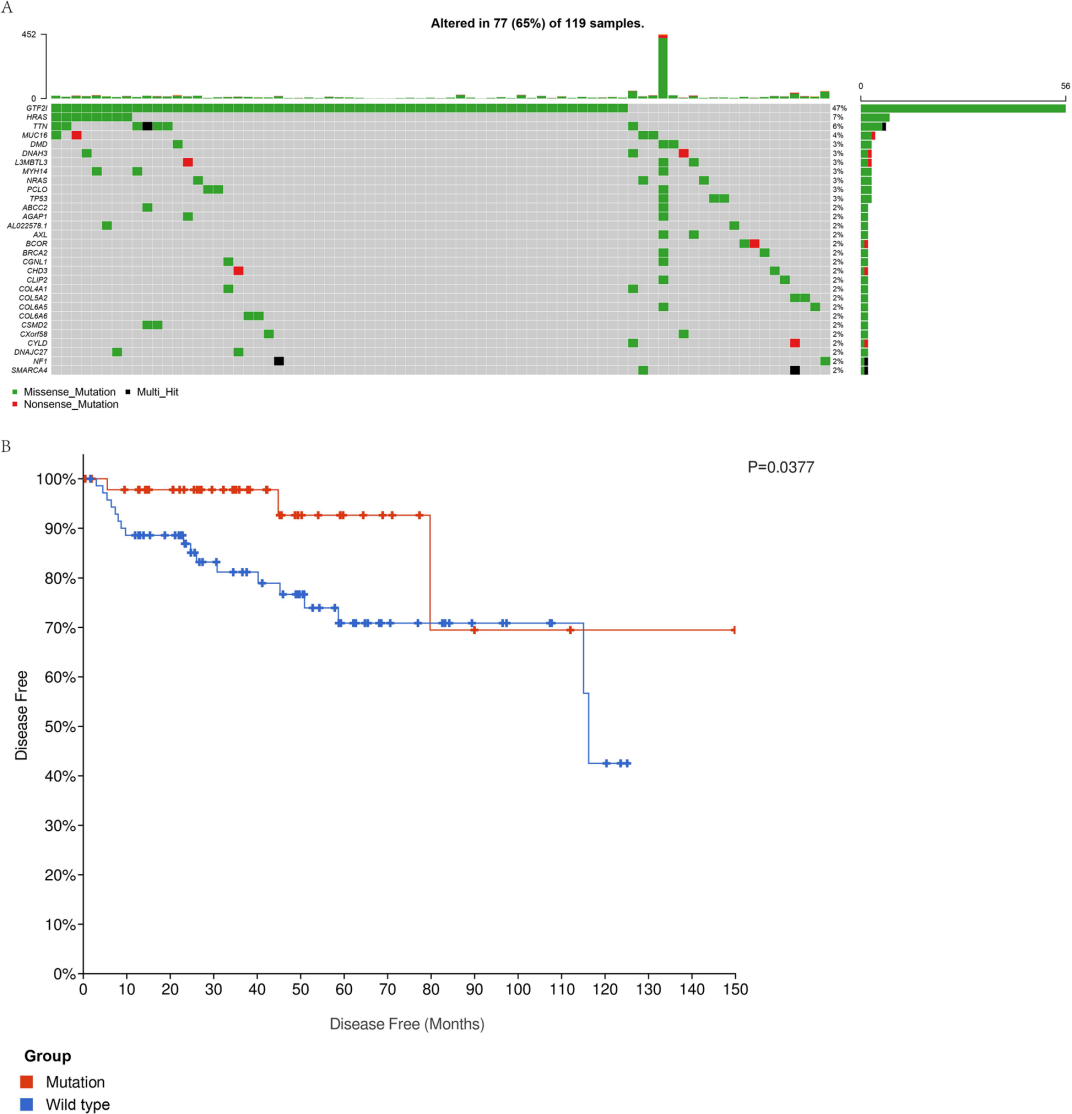
**A** Water plot of mutation profiles in each thymic epithelial tumors (TETs) sample. **B** Disease-free survival of patients with TET between GTF2I mutant and wild-type group

Gene mutations in all TET samples are shown in Fig. 1A, including gene mutation frequency and type. GTF2I (47%), muscle RAS oncogene homolog (MRAS) (7%), titin (TTN) (6%), and mucin 16 (MUC16) (4%) were four genes identified with the highest mutation frequency in the TET samples. Notably, GTF2I mutations were all missense mutations.

Based on the GTF2I mutations, we divided all TET patients into two groups: the GTF2I mutant and GTF2I wild-type. Survival curves showed that patients in the GTF2I mutant-type group had more favorable prognostic results with lower probabilities of tumor recurrence than the GTF2I wild-type group (Fig. 1B). The clinical features of the two groups are shown in Table 1. The GTF2I mutation was determined to be related to Masaoka stage I, more indolent pathological subtypes, and no history of myasthenia gravis (MG) or no radiotherapy treatment. GTF2I wild-type was associated with advanced pathological types, MG history, and radiotherapy treatment.

**Table 1:** Patient and tumor characteristics in the GTF2I mutant and wild-type group

Variables	GTF2I Mutation/ *N* = 56 (100%)	Wild type/ *N* = 63 (100%)	*p*-value
Gender			0.855
Female	26 (46.4)	31 (49.2)	
Male	30 (53.6)	32 (50.8)	
Age			0.232
Mean(±SD)	58.46(±13.32)	55.43(±14.13)	
Race			0.331
Hispanic or latino	3 (5.4)	7 (11.1)	
Not hispanic or latino	53 (94.6)	56 (88.9)	
MG histoty			0.003
Yes	8 (14.3)	27 (42.9)	
No	46 (82.1)	35 (55.6)	
Unknown	2 (3.6)	1 (1.6)	
Masaoka stage			0.039
I	23 (41.1)	13 (20.6)	
II	24 (42.9)	36 (57.1)	
III	4 (7.1)	11 (17.5)	
IV	3 (5.4)	3 (4.8)	
Unknown	2 (3.6)	0 (0)	
Pathologic type			< 0.001
Type A	14 (25.0)	2 (3.2)	
Type AB	27 (48.2)	8 (12.7)	
Type B1	3 (5.4)	11 (17.5)	
Type B2	7 (12.5)	24 (38.1)	
Type B3	3 (5.4)	9 (14.3)	
Thymic carcinoma	2 (3.6)	9 (14.3)	
Radiation therapy			0.004
Yes	10 (17.9)	27 (42.9)	
No	44 (78.6)	31 (49.2)	
Unknown	2 (3.6)	5 (7.9)	
Vital status			0.17
Dead	2 (3.6)	7 (11.1)	
Alive	54 (96.4)	56 (88.9)	

*Abbreviation*: *MG* myasthenia gravis

### Estimation of immune cell infiltration and immune checkpoints between GTF2I mutant and wild-type TETs

**Fig. 2:**
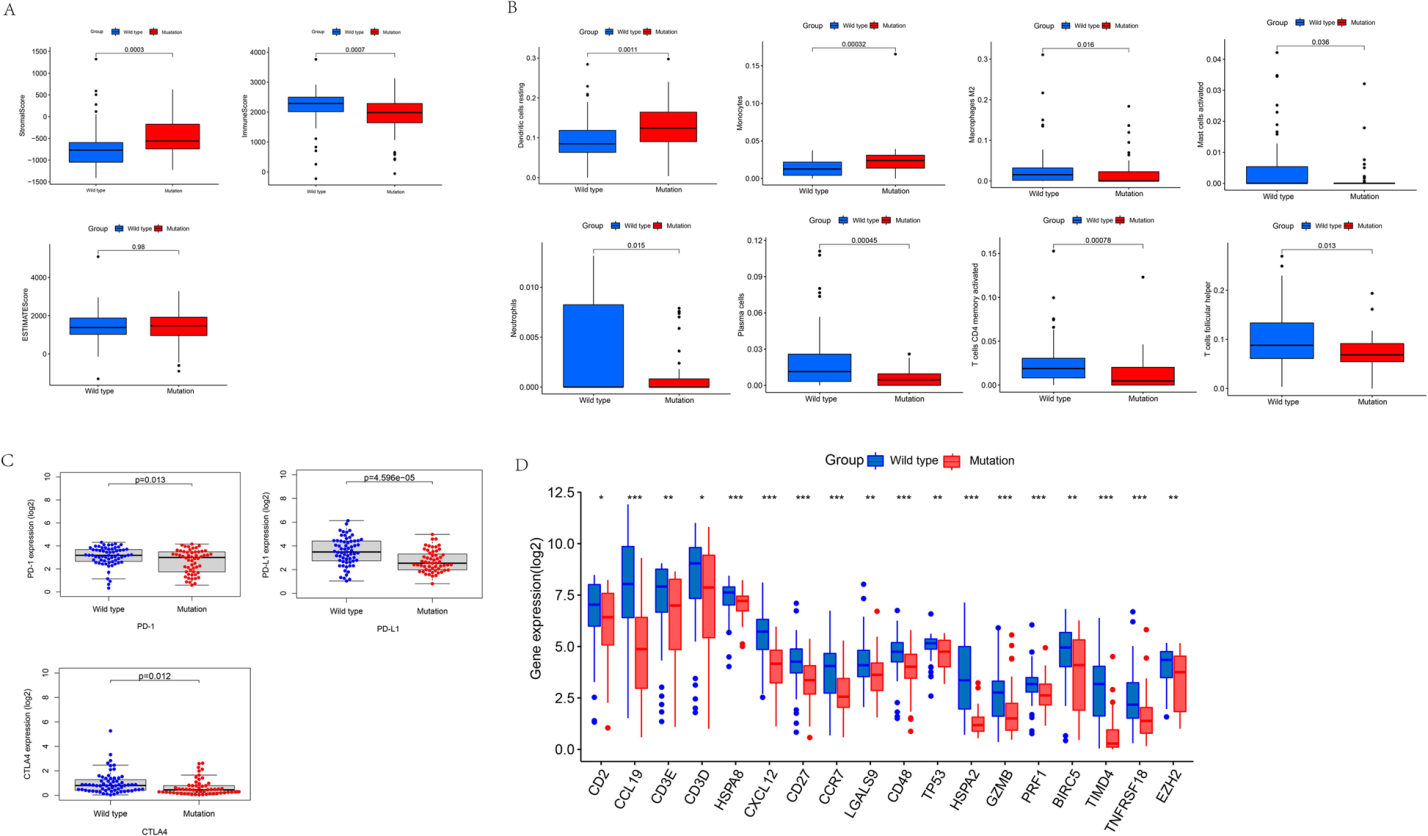
**A**-**B** The comparison of immune-related scores (**A**) and infiltrating levels of 22 immune cell types (**B**) between GTF2I wild type group and GTF2I mutant group. **C**-**D** The comparison of PD-1, PD-L1, CTLA4 (**C**), and 18 immune checkpoints expression (**D**) between GTF2I wild type group and GTF2I mutant group

There was no significant difference (*p* = 0.98) in estimate scores between the GTF2I mutant and wild-type groups (Fig. 2A). The stromal score and immune cell infiltrations with resting dendritic cells and monocytes in the GTF2I mutant group were higher than those in the GTF2I wild-type group (Fig. 2A-B). The immune score and immune cell infiltrations with M2 macrophages, activated mast cells, neutrophils, plasma cells, T helper follicular cells, and activated memory CD4 T cells in the GTF2I mutant group were lower than in the GTF2I wild-type group (Fig. 2A-B). The expression of PD-1, PD-L1, and CTLA4 in the GTF2I mutant group were lower than in the GTF2I wild-type group (Fig. 2C). Immune checkpoints such as CD2, C-C motif chemokine ligand 19 (CCL19), CD3 epsilon subunit of T-cell receptor complex (CD3E), heat shock protein family A (Hsp70) member 8 (HSPA8), CD3 delta subunit of T-cell receptor complex (CD3D), C-X-C motif chemokine ligand 12 (CXCL12), CD27, C-C motif chemokine receptor 7 (CCR7), galectin 9 (LGALS9), CD48, TP53, heat shock protein family A (Hsp70) member 2 (HSPA2), granzyme B (GZMB), perforin 1 (PRF1), baculoviral IAP repeat containing 5 (BIRC5), T cell immunoglobulin and mucin domain containing 4 (TIMD4), TNF receptor superfamily member 18 (TNFRSF18), and enhancer of zeste 2 polycomb repressive complex 2 subunit (EZH2) showed a higher level of expression in the GTF2I wild-type group than in the GTF2I mutant group (Fig. 2D).

### GSEA

The top nine pathways with enrichment in the GTF2I mutant group were the adherens junction, dilated cardiomyopathy, arrhythmogenic right ventricular cardiomyopathy, vascular smooth muscle contraction, basal cell carcinoma, melanogenesis, Notch signaling pathway, transforming growth factor (TGF) beta signaling pathway, and wingless/integrated (WNT) signaling pathway (Fig. . 3A).

**Fig. 3:**
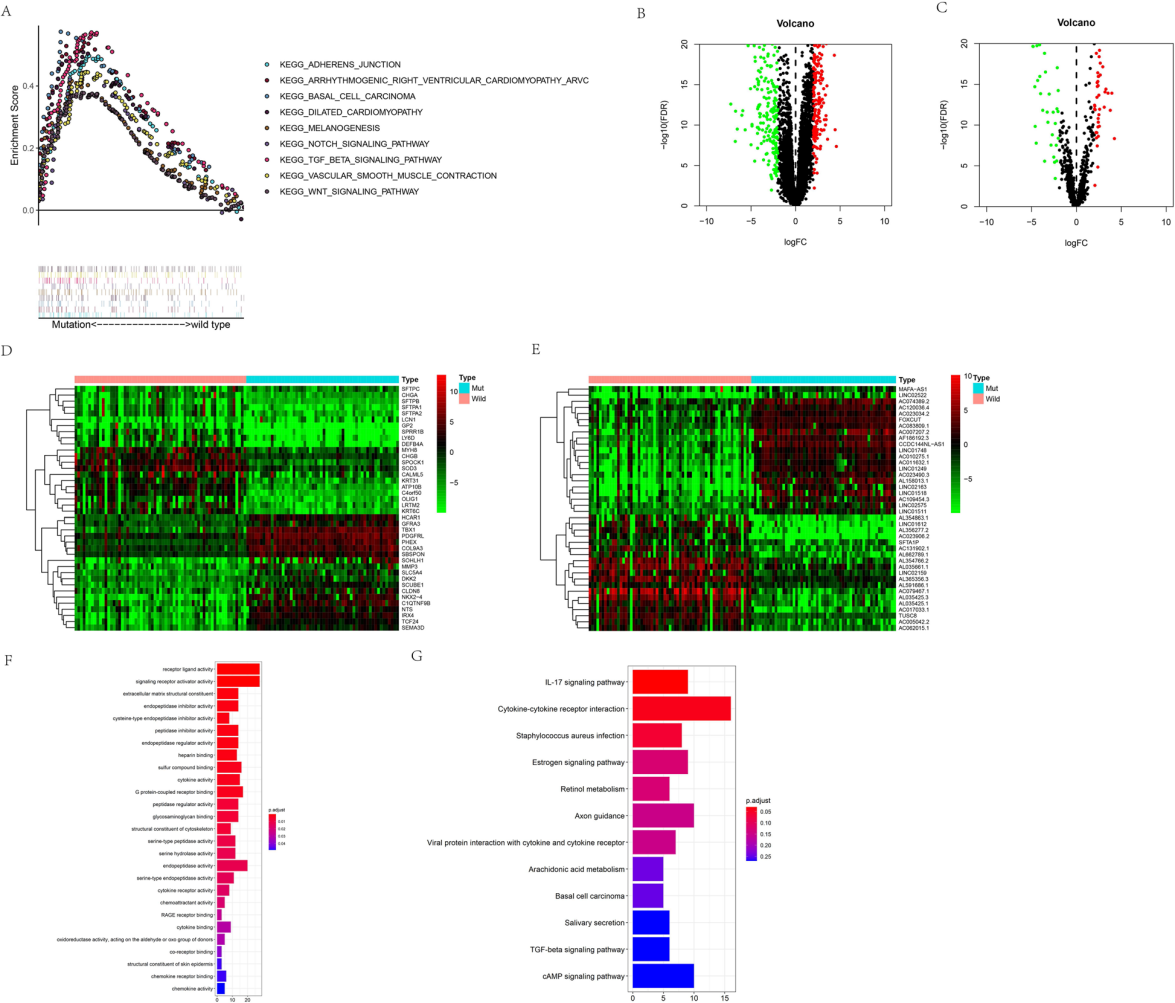
**A** GSEA results of KEGG analysis. **B**-**C** Volcano map of DEGs (**B**) and DERs (**C**) between GTF2I wild type group and GTF2I mutant group samples. **D**-**E** Heatmap of the top 40 genes (**D**) and lncRNAs (**E**) that were most significantly differentially expressed between GTF2I wild type group and GTF2I mutant group samples. **F**-**G** Barplots of GO analyses (**F**) and KEGG analyses (**G**)

### Functional enrichment analysis of DEGs

We performed differential expression analysis in the GTF2I mutant and wild-type TET samples, resulting in 396 differentially expressed genes (DEGs) and 93 differentially expressed lncRNAs (DERs) visualized as volcano plots (Fig. . 3B-C). The heatmaps showed the top 40 genes and lncRNAs that were the most significantly differentially (*p* < 0.05) expressed between GTF2I wild-type and mutant TETs (Fig. . 3D-E). Gene ontology (GO) analysis revealed that extracellular matrix structural constituent, endopeptidase, signaling receptor activator activity, endopeptidase inhibitor, and receptor-ligand activity, were significantly enriched (Fig. . 3F). The Kyoto encyclopedia of genes and genomes (KEGG) analysis demonstrated that the staphylococcus aureus infection, cytokine-cytokine receptor interaction, and IL-17 signaling pathway were significantly enriched (*p* < 0.05) (Fig. . 3G).

### Construction and validation of the risk assessment model

**Fig. 4:**
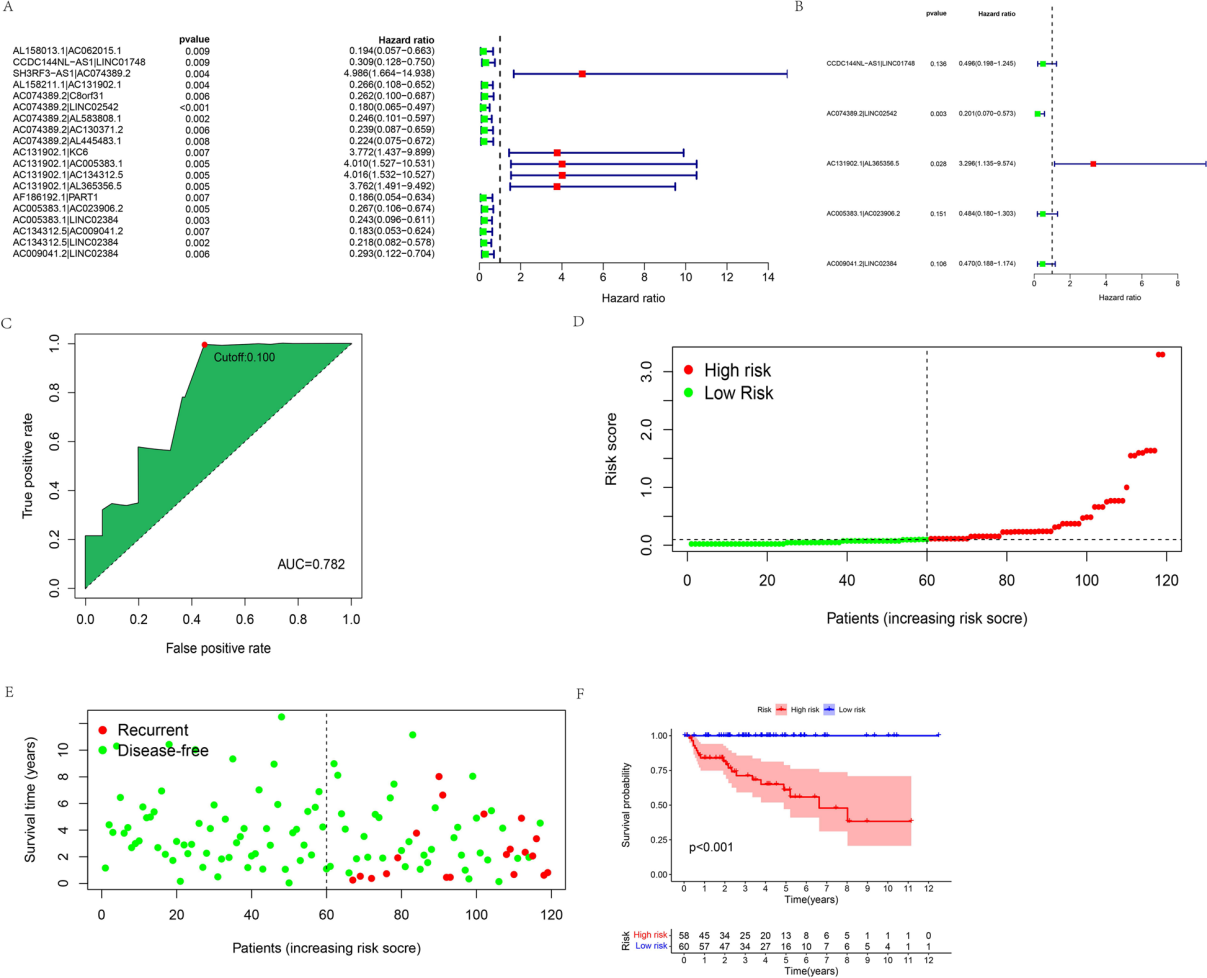
**A** The prognostic IRDER pairs extracted by univariate cox regression analysis. **B** The prognostic signature constructed by multivariate cox regression analysis. **C** AUC of the signature and the best cutoff value obtained by the AIC. **D** Risk score distribution of patients between high- and low-risk groups. **E** Tumor recurrent status of patients between high- and low-risk groups. **F** Kaplan–Meier survival curve of patients between high- and low-risk groups

**Fig. 5:**
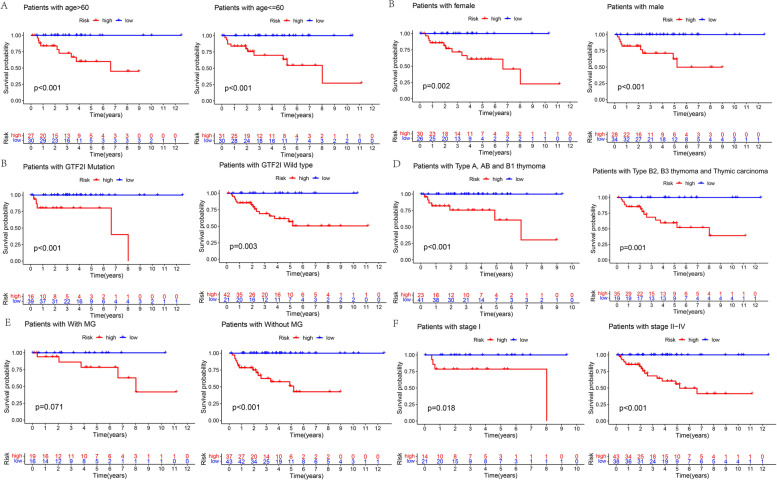
Kaplan–Meier survival curves stratified by age (**A**), gender (**B**), GTF2I mutation (**C**), histologic type (**D**), MG history (**E**) and Masaoka stage (**F**) between low- and high-risk groups

Eight immune-related differentially expressed genes (IRDEGs) and 53 immune-related differentially expressed lncRNAs (IRDERs) were identified by taking the intersection and performing Spearman’s correlation analysis. We used an iterative cycle among the 53 IRDERs and identified 1266 IRDER pairs. We performed univariate Cox regression analysis on all IRDER pairs and identified 19 IRDER pairs (Fig. 4A). Next, we performed multivariate Cox regression which resulted in five IRDER pairs to build the risk model (Fig. 4B). When the Akaike’s information criterion (AIC) value referred to 0.1, we identified the maximum knee point as the cut-off value and attained the largest area under the curve (AUC) value of 0.782 (Fig. 4C). The formula used to calculate the risk score for all TET patients was:

Risk score = − 0.700794392573432× CCDC144NL-AS1|LINC01748–1.60567899345149× AC074389.2|LINC02542+ 1.19279714426103× AC131902.1|AL365356.5–0.725120180351969× AC005383.1|AC023906.2–0.755363912198633× AC009041.2|LINC02384. We divided the groups into high- and low-risk using the cut-off value of 0.1 (Fig. 4D). We observed that patients with a high-risk score had a greater chance of tumor recurrence and a shorter time to recurrence (Fig. 4E). Kaplan-Meier analysis showed that patients in the low-risk group exhibited better prognostic results with lower odds of tumor recurrence than in the high-risk group (Fig. 4F). In addition, the clinicopathologic characteristics such as age (Fig. 5A), gender (Fig. 5B), GTF2I mutation (Fig. 5C), histologic subtype (Fig. 5D), MG history (Fig. 5E) and Masaoka stage (Fig. 5F) also demonstrated that patients in the low-risk group had lower odds of tumor recurrence compared with the high-risk group.

### Validation of the risk assessment model

**Fig. 6:**
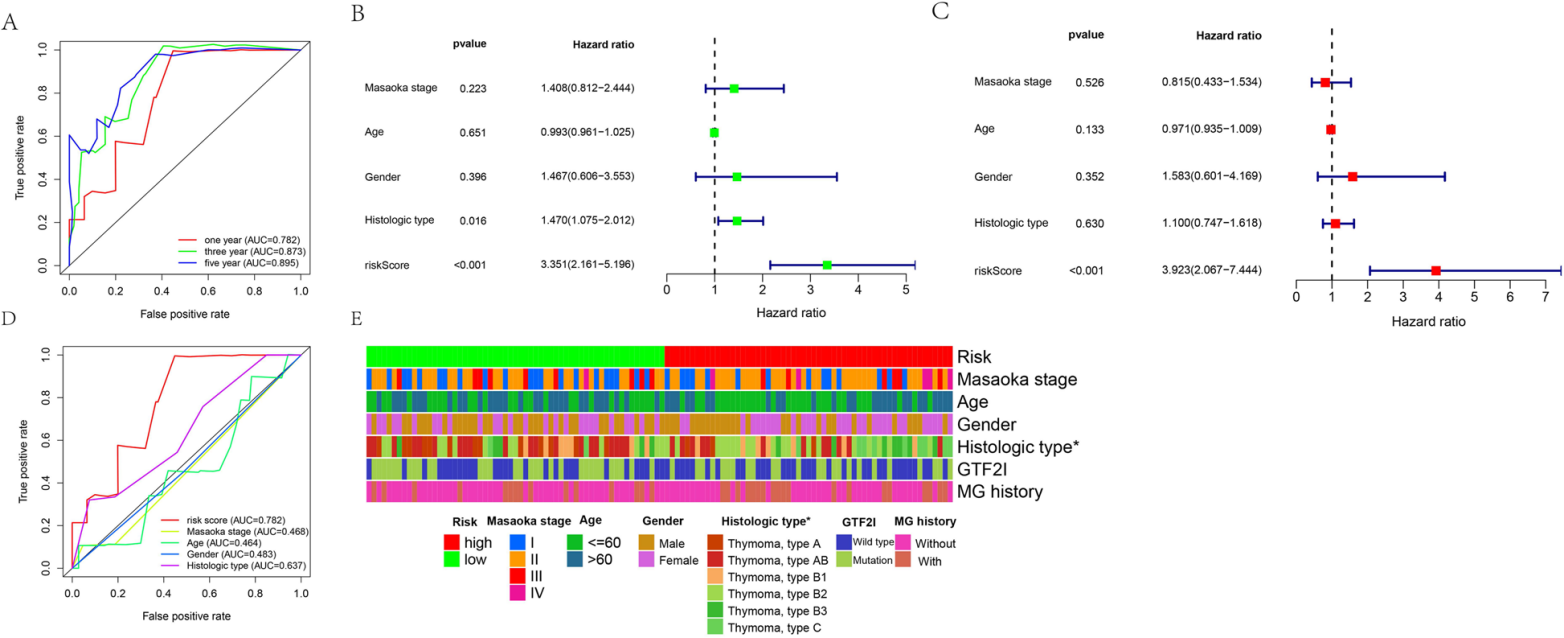
**A** Time-dependent ROC curve analyses of risk score. **B** Univariate Cox analyses of clinical factors and risk score. **C** Multivariate Cox analyses of clinical factors and risk score. **D** One-year ROC curve analyses of Masaoka stage, Age, Gender, Histologic type and risk score. **E** Heatmap showed the comparison of the relationship between the clinical characteristics of patients between high- and low-risk groups

**Fig. 7:**
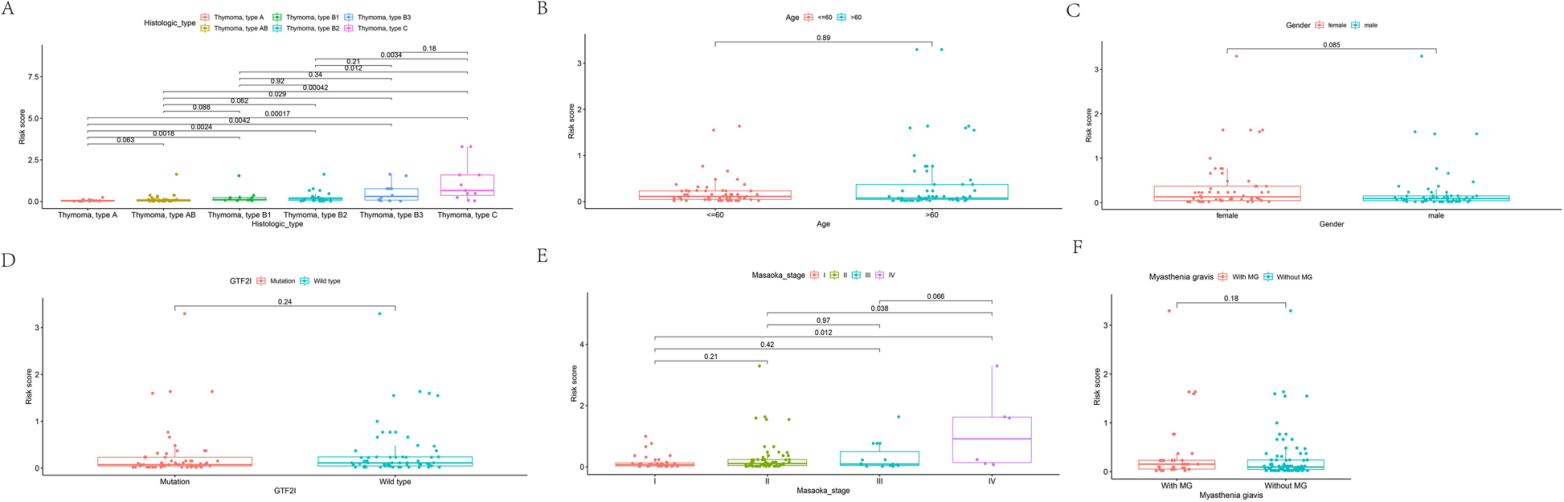
**A**-**F** The scatter diagram showed the relationship between histologic type (**A**), age (**B**), gender (**C**), GTF2I mutation (**D**), Masaoka stage (**E**), and MG history (**F**) and the risk score

Time-dependent receiver operating characteristic (ROC) curves were used to assess the specificity and sensitivity of the risk model and the 1-, 3-, and 5-year AUCs were 0.782, 0.873, and 0.895, respectively (Fig. 6A). Univariate Cox regression analysis showed that the hazard ratio (HR) of the histologic type and 95% confidence interval (CI) were 1.470 and 1.075–2.012 (*p* = 0.016), respectively. HR of the risk score and 95% CI were 3.351 and 2.161–5.196 (*p* < 0.001), respectively (Fig. 6B). Results of multivariate Cox regression analysis showed that risk score (HR = 3.923; 95% CI = 2.067–7.444; *p* < 0.001,) was an independent prognostic predictor (Fig. 6C). The AUC values for Masaoka stage, age, gender, and histologic subtype in 1-year survival were 0.468, 0.464, 0.483, and 0.637, respectively (Fig. 6D). The strip charts (Fig. 6E) and consequent scatter diagrams formed from a Wilcoxon signed-rank test revealed that a higher risk score was significantly related (*p* < 0.05) to more aggressive histologic subtypes (Fig. 7A). There was no significant association between the risk score and age (Fig. 7B), gender (Fig. 7C), GTF2I mutation (Fig. 7D), Masaoka stage (Fig. 7E), and MG history (Fig. 7F).

### Construction and validation of the nomogram

**Fig. 8:**
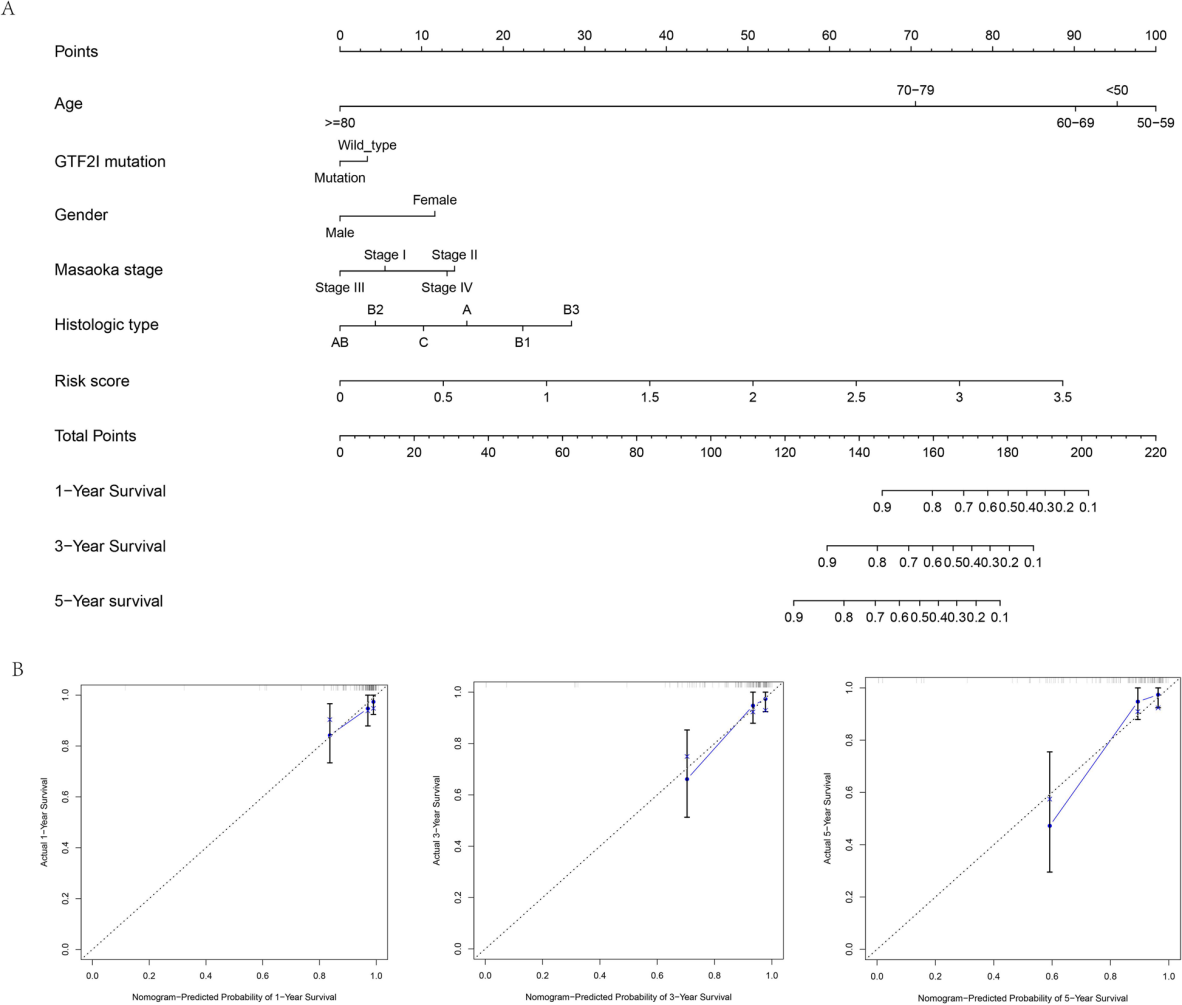
**A** The nomogram containing the risk score. **B** The calibration curves for 1-year, 3-year, and 5-year disease-free survival

A nomogram was established using risk score and common clinical factors of patients with TETs, including age, GTF2I mutation, gender, Masaoka stage, and histologic subtype (Fig. 8A). The 1-, 3-, and 5-year calibration curves showed a strong ability to predict tumor recurrence (Fig. 8B).

## Discussion

Through the analysis of thymoma mutation data from TCGA, we determined that GTF2I mutations were highly common in thymoma and were mostly missense mutations, which was consistent with previous findings [8, 9, 25, 26]. Additionally, we found that the GTF2I mutant type had a lower chance of tumor recurrence, and a longer disease-free survival than the wild-type TET. Previous studies have also shown that type B1, B2, B3, and thymic carcinoma were more aggressive pathological subtypes with a worse prognosis, whereas thymoma type A and type AB exhibited more favorable prognosis [4, 5]. Mutant type was more concentrated in thymoma type A and AB, whereas the wild-type occurred more often in type B1, B2, B3, and thymic carcinoma. This demonstrates to some extent that the prognosis relevant to GTF2I in TET provides a potential molecular mechanism for the prognosis relevant to pathological subtypes. However, some studies showed inconsistent results whereby more indolent subtypes of TETs, such as type A and AB thymomas, were more likely to recur [6].Further research is still needed to determine the intrinsic truth.

Due to the limited studies exploring the relationship between GTF2I mutations and TIME worldwide, we investigated the differences in the TIME between TET patients with GTF2I mutant thymoma and wild-type, with the aim to illustrate prognostic differences between the two groups. According to the TIME infiltration characterization of patients with mutant and wild-type TETs, we defined GTF2I mutant type as the most immune stable type because the peak of immune response passed and the immune response was more stable. We defined the GTF2I wild-type as the more immune stressed type because the immune response had not yet reached the peak, the immune response to tumor antigen stimulation was strong, the immune response continued to enhance in the development to the peak, and various immune checkpoints were highly expressed with great immunotherapy potential [27–29]. Including three star immune checkpoints, PD-1, PD-L1, and CTLA4, a total of 21 immune checkpoints were identified as upregulated in GTF2I wild-type TET samples in our study. Therefore, once we find that a patient with advanced TET is GTF2I wild-type is diagnosed, indicating that the patient has a poor prognosis, better efficacy may be achieved by using PD-1, PD-L1, or CTLA4 inhibitors for treatment. Some previous studies have shown that anti-PD-L1 or anti-PD-1 antibodies such as pembrolizumab and avelumab can have a therapeutic effect on recurrent and metastatic advanced thymoma or thymic carcinoma and can control tumor growth [30–32]. We recommend that patients with advanced TETs should be tested for GTF2I gene mutation status, to more accurately predict patient prognosis and predict immune checkpoint inhibitor responses.

For the GTF2I wild-type TETs, multiple immune cells were highly infiltrated. The immune response was in a state of continuous activation, which was too strong and would attack their own normal tissues and organs, hence, the patients were in a state of continuous stress. Therefore, GTF2I wild-type TET patients were more likely to form autoimmune diseases as well as develop symptoms of MG than patients with a mutant type. At the same time, our results suggested that the use of PD-1, PD-L1 or CTLA4 inhibitors in the treatment of patients with advanced TETs required close attention on the generation of immune-related adverse reactions such as MG while treating tumors. Anti-PD-L1 or anti-PD-1 antibodies have been reported to cause adverse reactions such as MG, myocarditis and pneumonia in patients with TETs [32–35]. Further, our study provides multiple potential immunotherapeutic targets such as CD2. The CD2 monoclonal antibody DANYELZA may be an option for TET patients if they exhibit low responsiveness or poor tolerability to PD-1 or PD-L1 inhibitors. Our study has identified immune checkpoints requiring further investigation.

The Notch, TGF beta and WNT signaling pathways were enriched in the GTF2I mutant group. Previous studies have reported that these signaling pathways play an important role in regulating TIME and cancer progression [36–41]. The results of GO and KEGG enrichment analyses illustrated that IRDEGs were closely related with cytokine receptor interactions, receptor-ligands interactions, and signaling receptor mutual activation.

Several studies have shown that lncRNAs could be used as prognostic marker in multiple cancers [42–46], and could build signatures that can predict tumor recurrence [47–49]. However, due to inconsistent accuracy when detecting lncRNA expression level, which could deeply influence clinical practice, we constructed a lncRNA signature using a novel lncRNA method that can reduce the errors caused by the detection platform [22–24]. To our knowledge, the establishment of the lncRNA pairs method to construct a predictive signature in TETs to predict the prognosis of TET patients before has not yet been conducted. Moreover, good efficacy of this signature in predicting the prognosis of TET patients further verifies the importance of GTF2I mutations in TETs. We also determined that some lncRNAs associated with GTF2I mutations may play an important role in TETs, which to our knowledge has not been previously reported. The lncRNA CCDC144NL-AS1 was significantly upregulated in gastric cancer tissues and was related to poor prognosis [50]. Overexpression of lncRNA CCDC144NL-AS1 has also been associated with poor prognosis of patients suffering from non-small cell lung cancer [51]. The lncRNA LINC01748 acts as an independent predictor of poor prognosis in non-small cell lung cancer patients [52]. Our study showed that the lncRNAs CCDC144NL-AS1 and LINC01748 may also play an important role in the development of thymoma and are prognostic markers requiring further investigation.

Despite the implications of our results, this study still has several limitations. The number of TET patient samples used to compare clinical features and TIME between GTF2I mutant-types and wild-types and used to establish the lncRNA pairs signature was small (119) and external validation was not available.

## Conclusion

We analysed TIME differences between patients with GTF2I mutant and wild-type TETs, and defined GTF2I mutant-type as immune stable and the GTF2I wild-type as immune stressed. In addition, we established a signature based on IRDEGs to predict tumor recurrence, and a risk score was determined as an independent clinical prognostic factor. Our study improves the understanding of GTF2I mutations in the TIME and provides more insight into effective immunotherapy strategies.

## Methods

### Data processing

We downloaded the tumor mutation and transcription data of 119 patients with TETs and their clinicopathological information from TCGA database, however, clinical information was missing from some patients. Three hundred and thirty-two immune related genes were obtained from the immune system process gene set (systemic name: M13664) in the Gene Set Enrichment Analysis (GSEA) (http://www.gsea-msigdb.org/gsea/index.jsp).

### Evaluation of gene mutation in TETs

Gene mutation in TETs was visualized using a waterfall plot with the R package “maftools” and oncoplot function. Differences such as gender, age, race, MG history, Masaoka stage, histologic type, radiation therapy history, and vital status were compared using a Chi-square test between GTF2I mutant and wild-type TET patients.

We performed Kaplan-Meier analysis to assess the disease-free survival difference between GTF2I mutant and wild-type TET patients and visualized this via survival curves using the “survival” and “survminer” R packages, and the ggsurvplot function.

### Investigation of the TIME and immune checkpoints

CIBERSORT (http://cibersort.stanford.edu/) can evaluate immune cell infiltration levels on the basis of the gene expression profiles from complex tissues [53]. Using the transcriptional profile of TETs and this software, we calculated the relative percentage of infiltration of 22 immune infiltrating cells and used wilcoxTest function, the “limma” and “ggpubr” R packages to assess the differences between GTF2I mutant and wild-type TET patients. We calculated the immune score of each sample using the R package “ESTIMATE” and analyzed the difference between GTF2I mutant and wild-type TET patients [54]. We identified 178 immune checkpoints by reviewing existing literatures. Then we performed gene expression differential analysis between GT2I mutant samples and wild-type samples, and obtained 65 differentially expressed immune checkpoint genes. Finally, we removed genes with a median immune checkpoint gene expression (log2) less than 1, and obtained 18 immune checkpoints.

### GSEA

We utilized gene sets (c2. cp. kegg.v6.2.-symbols) with GSEA software (https://www.gsea-msigdb.org/gsea/login.jsp) to investigate differences across pathway activity between GTF2I mutant and wild-types (*p* < 0.05).

### Differential expression analysis between GTF2I mutant and wild-type TET tissues and enrichment analysis of the differentially expressed genes

We identified the DEGs and lncRNAs by comparing the TET tissues of 56 patients with GTF2I mutation and 63 patients without GTF2I mutation from TCGA dataset with a threshold for false discovery rate of < 0.05, along with |log2 FC (fold-change) | > 2 using the R package “limma”. We performed KEGG and GO enrichment analyses of these DEGs using the R package “clusterProfiler”, “org.Hs.eg.db” and “enrichplot” to investigate the biological processes difference between GTF2I mutant and wild-type TETs.

### Pairing lncRNAs methods

We obtained IRDEGs by taking the intersection of immune genes and DEGs. Using Spearman’s correlation analysis between the IRDEGs and DERs, we received IRDERs with correlation coefficients > 0.4 and *p* < 0.05. We compared all IRDERs pairwise with ifelse function; in a lncRNA pair, one lncRNA was defined as a lncRNA a and the other is defined as lncRNA b. When lncRNA a was larger than lncRNA b, its value was defined as 1. When lncRNA a was smaller than lncRNA b, it was defined as 0.

### Construction of the lncRNA pairs prognostic risk signature

First, we performed a univariate Cox regression analysis with *p* < 0.01 in all IRDER pairs. Then, we performed multivariate Cox regression and identified five IRDER pairs to build the risk model for predicting TET recurrence. The formula used to calculate the risk score is as follows:

$$risk\ Score={\hat{h}}_0(t){\sum}_{i=1}^k\beta iSi,$$

where βi is the expression quantity of the IRDER pairs and Si is the coefficient of correlation of the IRDER pairs.

The maximum knee was determined by the AIC value of the 1-year ROC curve, which was used as a cut-off point of the risk score for classifying patients into the high- and low-risk groups. We used the R package “survivalROC” and polygon function to plot the 1-, 3-, 5-year ROC curves. We performed Kaplan-Meier analysis to assess the disease-free survival difference between the high- and low-risk groups and was visualized through survival curves by using the R packages “survival” and “survminer”, and ggsurvplot function. Univariate and multivariate Cox regression analyses were performed between risk score and clinical features such as age, gender, histologic type, Masaoka stage, and GTF2I mutation. We also conducted a Wilcoxon signed-rank test to compare the risk score differences between groups with different clinical features.

### Construction and validation of the nomogram

Common clinical features in patients with TETs, such as age, GTF2I mutation, gender, Masaoka stage, and histologic type, were used to construct a nomogram using the R package “rms” and calibrate function. The time-dependent calibration curves from the R package “rms” were used to determine prediction accuracy of the nomogram [55, 56].

## Data Availability

The entire RNA-seq profile data, mutation data and the clinical data of ESCC patients in this study come from The Cancer Genome Atlas (TCGA, https://cancergenome.nih.gov/) database.

## Abbreviations

AIC: Akaike’s information criterion
BIRC5: Baculoviral IAP repeat containing 5
CCL19: C-C motif chemokine ligand 19
CCR7: C-C motif chemokine receptor 7
CD3D: CD3 delta subunit of T-cell receptor complex
CD3E: CD3 epsilon subunit of T-cell receptor complex
CI: Confidence interval
CXCL12: C-X-C motif chemokine ligand 12
DEG: Differentially expressed gene
DER: Differentially expressed lncRNA
EZH2: Enhancer of zeste 2 polycomb repressive complex 2 subunit
GO: Gene Ontology
GSEA: Gene Set Enrichment Analysis
GTF2I: General transcription factor IIi
GZMB: Granzyme B
HR: Hazard ratio
HSPA2: Heat shock protein family A (Hsp70) member 2
HSPA8: Heat shock protein family A (Hsp70) member 8
IRDEG: Immune-related differentially expressed gene
IRDER: Immune-related differentially expressed lncRNA
KEGG: Kyoto Encyclopedia of Genes and Genomes
LGALS9: Galectin 9
LncRNA: Long non-coding RNA
MG: Myasthenia gravis
MRAS: Muscle RAS oncogene homolog
MUC16: Mucin 16
PRF1: Perforin 1
ROC: Receiver operating characteristic
TCGA: The Cancer Genome Atlas
TET: Thymic epithelial tumor
TGF: Transforming growth factor
TIMD4: T cell immunoglobulin and mucin domain containing 4
TIME: Tumor immune microenvironment
TNFRSF18: TNF receptor superfamily member 18
TTN: Titin
WHO: World Health Organization
WNT: Wingless/integrated
